# Robotic Versus Video-Assisted Thoracoscopic Lobectomy/Segmentectomy: Multilevel Analysis in Japan

**DOI:** 10.1093/icvts/ivag005

**Published:** 2026-01-09

**Authors:** Yukiko Nemoto, Makoto Okawara, Natsumasa Nishizawa, Masataka Mori, Masaru Takenaka, Koji Kuroda, Yoshihisa Fujino, Shinya Matsuda, Kiyohide Fushimi, Fumihiro Tanaka

**Affiliations:** Second Department of Surgery, University of Occupational and Environmental Health, 1-1 Iseigaoka, Yahatanishi-ku, Kitakyushyu-shi, Fukuoka, 807-8555, Japan; Department of Preventive Medicine and Community Health, School of Medicine, University of Occupational and Environmental Health, 1-1 Iseigaoka, Yahatanishi-ku, Kitakyushyu-shi, Fukuoka, 807-8555, Japan; Second Department of Surgery, University of Occupational and Environmental Health, 1-1 Iseigaoka, Yahatanishi-ku, Kitakyushyu-shi, Fukuoka, 807-8555, Japan; Second Department of Surgery, University of Occupational and Environmental Health, 1-1 Iseigaoka, Yahatanishi-ku, Kitakyushyu-shi, Fukuoka, 807-8555, Japan; Second Department of Surgery, University of Occupational and Environmental Health, 1-1 Iseigaoka, Yahatanishi-ku, Kitakyushyu-shi, Fukuoka, 807-8555, Japan; Second Department of Surgery, University of Occupational and Environmental Health, 1-1 Iseigaoka, Yahatanishi-ku, Kitakyushyu-shi, Fukuoka, 807-8555, Japan; Department of Environmental Epidemiology, Institute of Industrial Ecological Sciences, University of Occupational and Environmental Health, 1-1 Iseigaoka, Yahatanishi-ku, Kitakyushyu-shi, Fukuoka, 807-8555, Japan; Department of Nursing, Fukuoka International University of Health and Welfare, 3-6-40 Momochihama, Sawara-ku, Fukuoka-shi, Fukuoka, 814-0001, Japan; Department of Health Policy and Informatics, Institute of Science Tokyo Graduate School of Medical and Dental Sciences, 1-5-45 Yushima, Bunkyo-ku, Tokyo, 113-8519, Japan; Second Department of Surgery, University of Occupational and Environmental Health, 1-1 Iseigaoka, Yahatanishi-ku, Kitakyushyu-shi, Fukuoka, 807-8555, Japan

**Keywords:** non-small-cell lung cancer, perioperative outcome, Japanese Diagnosis Procedure Combination database, robotic-assisted lobectomy, video-assisted lobectomy

## Abstract

**Objectives:**

Large-scale comparative data on the perioperative safety of robotic-assisted thoracoscopic surgery and video-assisted thoracoscopic surgery in Asia are limited. We compared the perioperative outcomes of these 2 approaches for lung cancer.

**Methods:**

This retrospective study used data from the Diagnostic Procedure Combination database in Japan. We included 47 541 patients who underwent lobectomy or segmentectomy for lung cancer from 2018 to 2021 and performed multivariable analyses.

**Results:**

Among 47 541 patients, 2835 underwent robotic-assisted thoracoscopic surgery. Perioperative mortality did not differ significantly between groups (incidence rate ratio, 1.71; 95% CI, 0.88-3.33). Robotic-assisted surgery was associated with longer anaesthesia time and a higher incidence of mechanical ventilation postoperatively (incidence rate ratio, 1.96; 95% CI, 1.36-2.81), although the absolute difference was small (Marginal risk difference, +0.52 percentage points; 95% CI, +0.14 to +0.91). No significant differences were observed in other major complications, reoperation, or hospital stay.

**Conclusions:**

In this large, real-world Japanese cohort including the early experience with robotic surgery, overall perioperative safety was comparable between robotic-assisted and video-assisted thoracoscopic surgery, although a statistically significant but small absolute increase in postoperative ventilation was observed with the robotic approach. This association remained robust across a series of sensitivity analyses. However, it is likely influenced by unmeasured confounding. Future prospective studies should investigate specific procedural factors, including anaesthetic management and patient selection, to optimize outcomes.

## Graphical abstract

**Figure:**
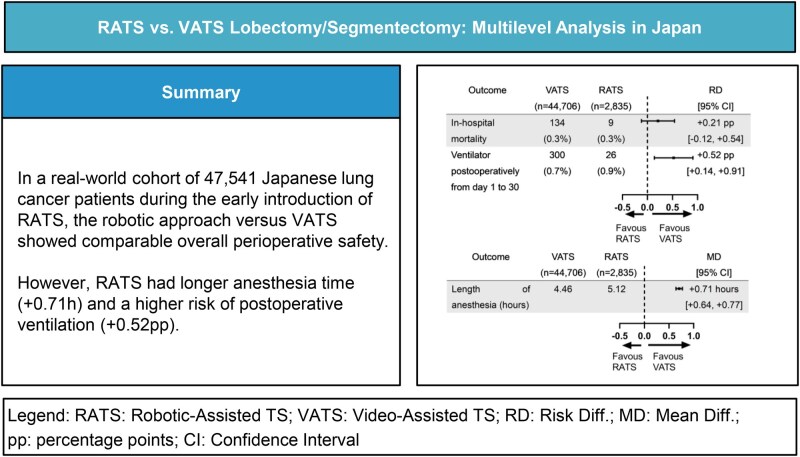

## INTRODUCTION

Video-assisted thoracoscopic surgery (VATS) and robotic-assisted thoracoscopic surgery (RATS) significantly improve short-term outcomes compared with traditional thoracotomy while maintaining equivalent long-term results.^1^ RATS offers potential technical advantages over VATS, including enhanced 3D visualization and superior instrument manoeuvrability, potentially leading to more precise dissection and a lower conversion rate to thoracotomy.^2^^,^^3^

In Japan, RATS for lung cancer was first covered by insurance for thoracoscopic lobectomies in April 2018. In 2020, thoracoscopic segmentectomies were covered by insurance.

Several randomized trials and meta-analyses have reported RATS to be comparable to VATS.^3–5^ Large-scale data comparing perioperative safety between VATS and RATS are lacking in Japan or Asia.

We compared the perioperative outcomes of lobectomy and segmentectomy using VATS and RATS in Japan. We conducted a multilevel analysis using real-world data, including hospitals with low hospital volume and caseload, using the Diagnostic Procedure Combination (DPC) database based on the health insurance system, covering the entire population of Japan.

## PATIENTS AND METHODS

The DPC database is a national administrative database established for research purposes under the guidance of the Japanese government. Its establishment and ongoing use are overseen by the DPC Research Group, ensuring consistency with Japan’s “Ethical Guidelines for Medical and Health Research Involving Human Subjects,” which aligns with the principles of the WMA Declaration of Taipei. For the present study, the use of this anonymized, pre-existing database was approved by the Institutional Review Board of the University of Occupational and Environmental Health, Japan (Approval Date: October 11, 2022, No: R4-045), which waived the requirement for individual patient consent due to the use of anonymized administrative data. The study was conducted in accordance with the principles of the Declaration of Helsinki.

### Study design (DPC database)

This retrospective, observational study used DPC data from the DPC Research Group of hospitalized patients in Japan.^6^ The DPC Research Group, a government-funded academic organization, independently collected electronic DPC data for research. Japan has a universal health insurance system; therefore, once a diagnosis is made, every citizen receives a standard level of medical care. Between 2018 and 2021, over 1700 hospitals used the DPC system, covering 85% of acute care general beds in Japan.^7^

### Patient cohort

We analysed data from 23 104 129 patients hospitalized in DPC system hospitals between April 1, 2018, and March 31, 2021. The inclusion criteria were a diagnosis of primary lung cancer (ICD-10 code: C34), age ≥18 years, and hospitalized patients who underwent lobectomy or segmentectomy. These procedures were identified using the Japanese medical intervention classification master code for lung resection (K514-2). Patients were then categorized into the VATS or RATS group based on specific health insurance billing codes: for lobectomy, VATS (150358810) and RATS (150406110); for segmentectomy, VATS (150358710) and RATS (150414410). The exclusion criteria were missing information (resection site, tumour node metastasis [TNM] classification, body mass index (BMI), activities of daily living, anaesthesia duration, and smoking history) and multiple lung malignancy surgeries during the same hospitalization.

### Endpoints

The primary endpoint was in-hospital mortality, defined as death from any cause during hospitalization, when surgery was performed. The secondary endpoints were other surgical outcomes, including anaesthesia time, duration of postoperative thoracic drainage and hospital stay, complications, reoperation under general anaesthesia for postoperative complications, postoperative pain, mechanical ventilation postoperatively from day 1 to 30 (defined as post-extubation re-initiation of non-invasive and invasive ventilation), postoperative interventions (thoracentesis and thoracic drainage), intraoperative blood transfusion, blood transfusion within 7 days postoperatively, and antibiotic use between days 5 and 14. The specific ICD-10 and Japanese medical intervention codes are listed in the **Supplementary Appendix**.

### Statistical analysis

We analysed the association between RATS or VATS and postoperative outcomes using bivariable and multivariable models. We used multilevel Poisson regression with robust variance for binary outcomes and multilevel linear regression for continuous variables. We selected Poisson regression to appropriately estimate effect sizes for high-incidence binary outcomes. To address overdispersion, a robust variance method using a sandwich estimator was employed.^8^ We applied random-intercept models, avoiding random-slope models due to complexity and convergence instability. We used age- and sex-adjusted analysis as a bivariable model. Patient factors that might be associated with postoperative outcomes were included as multivariable model covariates: sex, age, BMI, smoking history, disease stage, surgical procedure (lobectomy, segmentectomy), surgical site (right upper lobe, right middle lobe, right lower lobe, left upper lobe, left lower lobe), year of surgery, comorbidities (**Supplementary Appendix**), and number of lobectomy or segmentectomy procedures performed at each hospital (hospital level was divided into 4 categories according to the number of procedures, adjusted for differences in patient numbers). Hospital units were used as the category for random effects, and each hospital was assigned a unique hospital code.

We also calculated the absolute difference for each outcome. The estimand was the average treatment effect on the risk scale and mean scale: the marginal risk difference (RD) for each binary postoperative outcome and the marginal mean difference (MD) for each continuous outcome. Identification relied on conditional exchangeability (given measured covariates and hospital), positivity, and consistency/SUTVA.

For binary outcomes, we used mixed-effects models with a random intercept for hospital and obtained marginal risks (%) via g-computation (standardization) by averaging predicted risks over the observed covariate distribution, applying the log-normal moment correction (adding half of the random-intercept variance to the linear predictor). For continuous outcomes, we used mixed-effects linear models, computed marginal means from the fixed-effects linear predictor, and averaged them over the covariate distribution.

Effect measures were expressed as RD (percentage points [pp]; sign = RATS−VATS) and MD (original units; sign = RATS−VATS). 95% CIs were derived using the delta method.

Under these assumptions, the RD represents the standardized population-average change in events per 100 patients had the entire cohort received RATS rather than VATS. The MD represents the corresponding population-average change in the mean of the continuous outcome.

Stata Statistical Software Release 18 (StataCorp LLC, College Station, TX, United States) was used for statistical analyses. We considered *P* < .05 to indicate statistical significance.

### Sensitivity analysis

1. Endpoint definition: We re-evaluated the ventilator outcome using alternative definitions: (1) a shorter time window (ventilation within 1-7 postoperative days) and (2) a more specific event type (re-initiation of invasive ventilation only within 1-30 postoperative days).
2. Potential mediators: For ventilator outcomes, the analgesic method was considered a potential mediator. We additionally adjusted for potential mediators (nerve block, epidural anaesthesia, anaesthesia hours) to account for the influence of analgesic technique.
3. Temporal confounding: To account for potential temporal confounding, particularly from the early COVID-19 era, an analysis stratified by year of surgery was performed.
4. Learning curve: To account for the learning curve, we performed a sensitivity analysis excluding the first 20 RATS cases from each institution, a threshold supported by previous studies.^9–11^
5. Institutional clustering: To consider institutional differences in postoperative management, we examined between-institution variance (τ^2^) in the primary analysis and conducted an additional analysis restricted to facilities that performed both VATS and RATS during the study period.
6. Surgical selection bias: To account for potential bias in surgical selection, separate analyses were conducted for lobectomy and segmentectomy. Due to sparse data in the segmentectomy group, some outcomes did not occur; therefore, Firth-penalized logistic regression was applied as an alternative.
7. Missing data: Missing data are thought to result from omissions in the entry of inpatient information and are not dependent on any value such as patient severity. Therefore, the missing data in this study are considered to be either completely at random or at random.^12^ To address potential bias due to missing data, we performed multiple imputation using chained equations (30 imputations). After imputation, multilevel Poisson regression was repeated, and estimates were combined across imputations using Rubin’s rule. Because mixed-effects Poisson regression is not officially supported by “mi estimate” command in Stata, we used the “cmdok” option to combine estimates across 30 multiply imputed datasets. This approach provides valid pooled point estimates and standard errors under large-sample approximation, although degrees of freedom are not explicitly computed.
8. Covariate imbalance: To mitigate covariate imbalance, we applied inverse probability weighting (IPW)-weighted Poisson regression. Propensity scores for assignment to RATS versus VATS were estimated using logistic regression including the same covariates as in the main analysis, except for the year of surgery. Because assignments to VATS or RATS depended on the year and whether the hospital had introduced RATS, the analysis population was restricted to the final year of observation and limited to hospitals with both VATS and RATS cases during the observation period. To mitigate the influence of extreme values, values exceeding the 99th percentile were replaced with the 99th percentile using stabilized weights.

## RESULTS

### Patient characteristics

**Figure 1.**
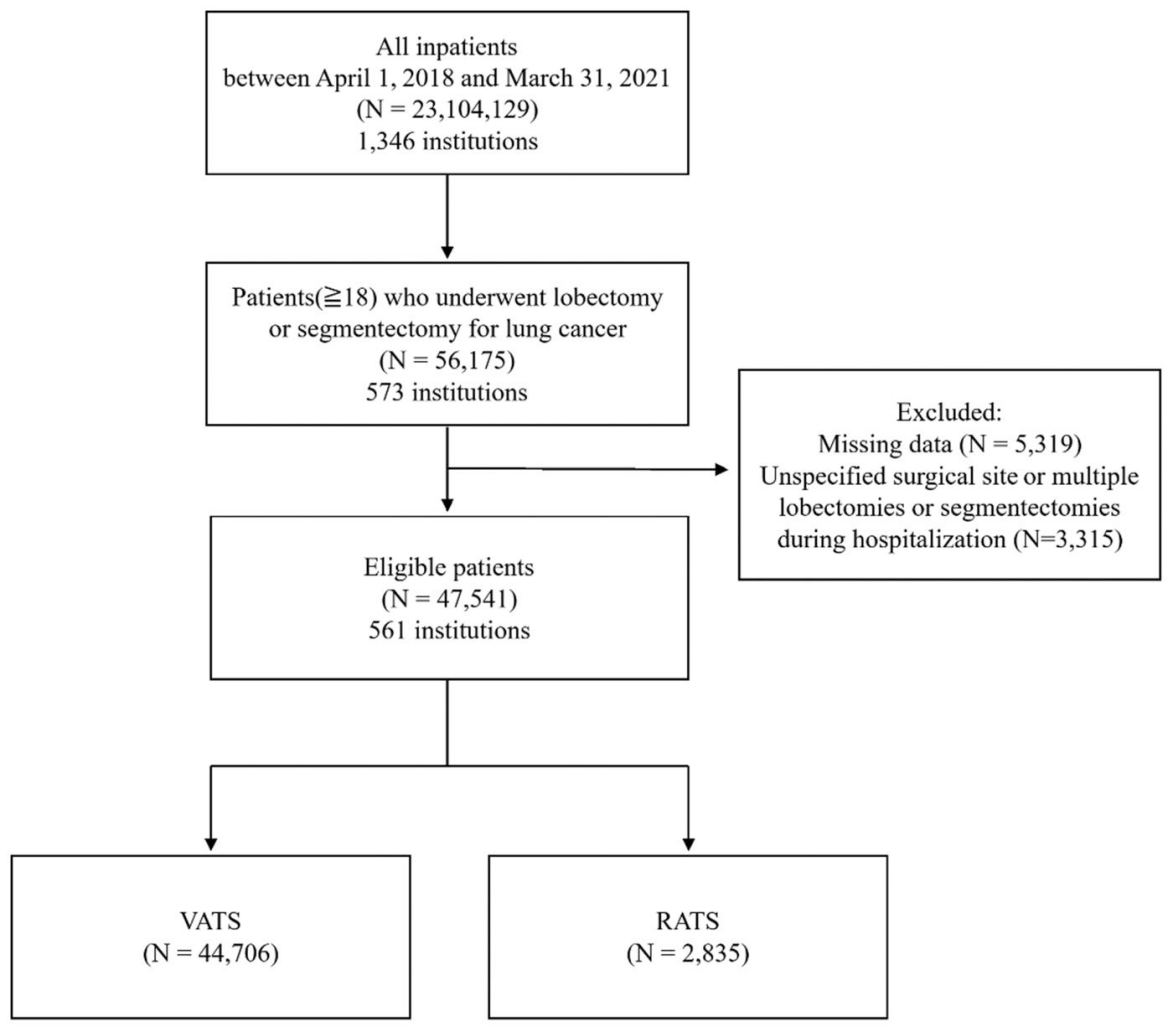
Flowchart Demonstrating the Patient Cohort From the DPC Database Included in the Study Analysis. Abbreviation: DPC, diagnostic procedure combination.

The number of patients included in the DPC database between April 1, 2018, and March 31, 2021, was 56 175 from 561 facilities. We excluded 8634 patients with missing data, multiple lobectomies, or segmentectomies during hospitalization. Ultimately, 47 541 patients were divided according to the surgical method used: VATS (44 706 patients) and RATS (2835 patients) (**Figure 1**).

### Changes in operative trends

In Japan, 15 911 cases of minimally invasive tissue sampling (RATS or VATS) were reported in 2018; 16 189 in 2019; and 15 441 in 2020. In 2018, 396 RATS cases, accounting for 2.5% of all surgeries, were reported. In 2019, 909 RATS cases, accounting for 5.6% of all the surgeries, were reported. In 2020, 1530 RATS cases were reported, accounting for 9.9% of all surgeries. The number and rate of RATS implementations have increased annually (**Figure S1**).

### Basic characteristics of study participants

The RATS group had a better smoking history, a less advanced TNM clinical stage, and fewer comorbidities. Lobectomy was performed more frequently in the RATS group. While 15.6% of patients in the VATS group underwent segmentectomy, only 5.6% of patients in the RATS group did (**Table 1**).

**Table 1.** Basic Characteristics of the Study Population

	Total	VATS	RATS
	*N* = 47 541	*N* = 44 706	*N* = 2835
Variables	*N* (%)	*N* (%)	*N* (%)
Sex, men	27 508 (57.9%)	26 023 (58.2%)	1485 (52.4%)
Age, mean (SD) (years)	70.3 (9.1)	70.4 (9.1)	69.3 (9.2)
18-59	5518 (11.6%)	5121 (11.5%)	397 (14.0%)
60-74	25 421 (53.5%)	23 841 (53.3%)	1580 (55.7%)
>75	16 602 (34.9%)	15 744 (35.2%)	858 (30.3%)
Body mass index (kg/m^2^)			
<18	1858 (3.9%)	1767 (4.0%)	91 (3.2%)
18-25	31 080 (65.4%)	29 174 (65.3%)	1906 (67.2%)
>25	14 603 (30.7%)	13 765 (30.8%)	838 (29.6%)
Smoking history (current or former)	28 004 (58.9%)	26 488 (59.2%)	1516 (53.5%)
Activities of daily living			
Independent	46 667 (98.2%)	43 874 (98.1%)	2793 (98.5%)
Walks with the help of one person	530 (1.1%)	500 (1.1%)	30 (1.1%)
Wheelchair user	210 (0.4%)	204 (0.5%)	6 (0.2%)
Total assistance	134 (0.3%)	128 (0.3%)	6 (0.2%)
Cancer TNM stage			
0	2625 (5.5%)	2422 (5.4%)	203 (7.2%)
I	36 243 (76.2%)	33 905 (75.8%)	2338 (82.5%)
II	5543 (11.7%)	5334 (11.9%)	209 (7.4%)
III	2739 (5.8%)	2665 (6.0%)	74 (2.6%)
IV	391 (0.8%)	380 (0.8%)	11 (0.4%)
Type of surgery			
Segmentectomy	7119 (15.0%)	6959 (15.6%)	160 (5.6%)
Lobectomy	40 422 (85.0%)	37 747 (84.4%)	2675 (94.4%)
Area of surgery			
Left upper	10 848 (22.8%)	10 367 (23.2%)	481 (17.0%)
Left lower	7361 (15.5%)	6963 (15.6%)	398 (14.0%)
Right upper	15 533 (32.7%)	14 414 (32.2%)	1119 (39.5%)
Right middle	3278 (6.9%)	3038 (6.8%)	240 (8.5%)
Right lower	10 521 (22.1%)	9924 (22.2%)	597 (21.1%)
Comorbidities			
Any of the following applies	25 023 (52.6%)	23 836 (53.3%)	1187 (41.9%)
Chronic obstructive pulmonary disease	5945 (12.5%)	5716 (12.8%)	229 (8.1%)
Interstitial pneumonia	1772 (3.7%)	1726 (3.9%)	46 (1.6%)
Pulmonary hypertension	36 (0.1%)	33 (0.1%)	3 (0.1%)
Respiratory insufficiency	189 (0.4%)	180 (0.4%)	9 (0.3%)
Chronic kidney disease	921 (1.9%)	879 (2.0%)	42 (1.5%)
Hypertension	12 388 (26.1%)	11 806 (26.4%)	582 (20.5%)
Diabetes	9172 (19.3%)	8700 (19.5%)	472 (16.6%)
Coronary artery disease	3155 (6.6%)	3029 (6.8%)	126 (4.4%)
Heart failure	1742 (3.7%)	1649 (3.7%)	93 (3.3%)
Arrhythmia	2143 (4.5%)	2027 (4.5%)	116 (4.1%)
Cerebral infarction	1941 (4.1%)	1863 (4.2%)	78 (2.8%)
Peripheral vascular disease	416 (0.9%)	394 (0.9%)	22 (0.8%)

Data are presented as *N* (%) or mean (SD).

Abbreviations: RATS, robotic-assisted thoracoscopic surgery; TNM, tumour node metastasis; VATS, video-assisted thoracoscopic surgery.

### Postoperative outcomes

**Figure 2.**
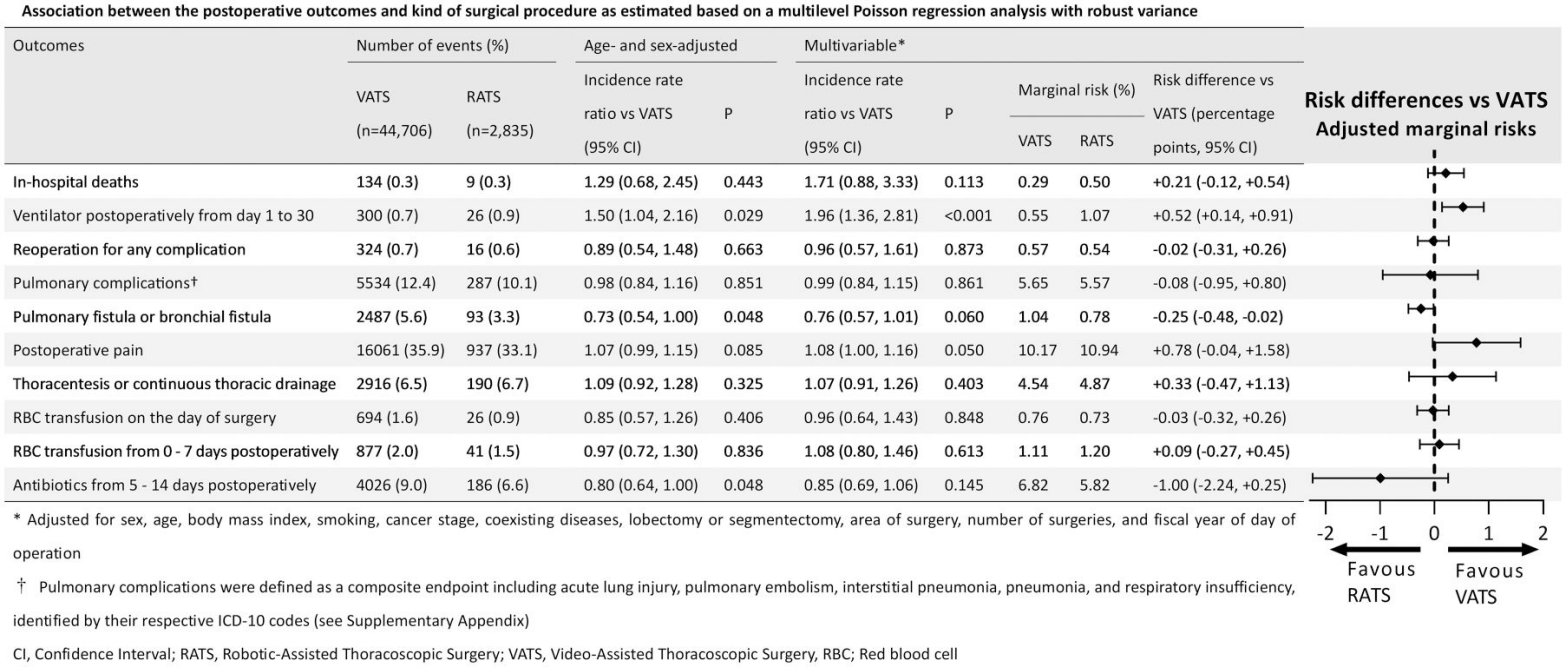
Association Between the Postoperative Outcomes and Kind of Surgical Procedure as Estimated Based on a Multilevel Poisson Regression Analysis With Robust Variance. Abbreviations: RATS, robotic-assisted thoracoscopic surgery; RBC, red blood cell; VATS, video-assisted thoracoscopic surgery.

**Table S1** shows the association between postoperative outcomes and surgical techniques. **Figure 2** presents the association between postoperative outcomes and surgical procedures based on multilevel Poisson regression with robust variance. We conducted bivariable analysis using age and sex as well as multivariable analysis. In-hospital mortality rates were 0.30% for the VATS group (134/44 706) and 0.32% for the RATS group (9/2835). The incidence rate ratio (IRR) for RATS compared with VATS was 1.29 (95% CI, 0.68-2.45) in the age- and sex-adjusted analysis and 1.71 (95% CI, 0.88-3.33) in multivariable analysis; however, the differences were not statistically significant. Marginal risk was 0.29% under VATS and 0.50% under RATS. RD was +0.21 pp (95% CI, −0.12 to +0.54), not significant.

For reoperation, postoperative pulmonary complications, and others, IRRs showed no significant differences between groups in either bivariable or multivariable analysis. However, in multivariable analysis, IRR was 0.76 (95% CI, 0.57-1.01) and 0.85 (95% CI, 0.69-1.06), respectively, with no significant difference. Marginal risk for “Pulmonary fistula or bronchial fistula” was 1.04% under VATS and 0.78% under RATS, and RD was −0.25 pp (95% CI, −0.48 to −0.02), significant. For “antibiotic administration on 5-14 days postoperatively,” marginal risk was 6.82% under VATS and 5.82% under RATS. RD was −1.00 pp (95% CI, −2.24 to +0.25), not significant. Mechanical ventilation postoperatively from day 1 to 30 was required in 0.67% of the VATS group (300/44 706) and 0.92% of the RATS group (26/2835). IRR was 1.50 (95% CI, 1.04-2.16) in the age- and sex-adjusted analysis and 1.96 (95% CI, 1.36-2.81) in multivariable analysis, indicating a significantly higher risk for RATS. The marginal risk was 0.55% under VATS and 1.07% under RATS, and RD was +0.52 pp (95% CI, +0.14 to +0.91), which was also significant.

**Figure 3.**
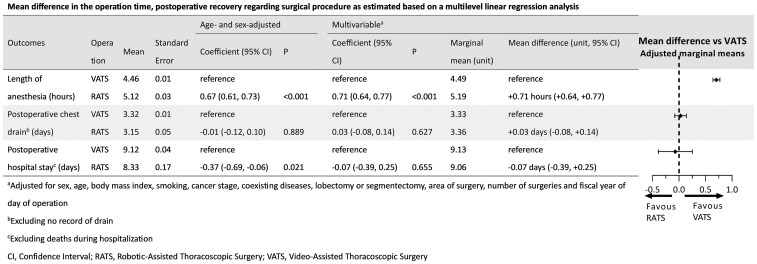
Mean Difference in the Operation Time, Postoperative Recovery Regarding Surgical Procedure as Estimated Based on a Multilevel Linear Regression Analysis. Abbreviations: RATS, robotic-assisted thoracoscopic surgery; VATS, video-assisted thoracoscopic surgery.

**Figure 3** presents the mean differences in general anaesthesia duration, postoperative chest tube placement duration, and postoperative hospital stay, as estimated using multistage linear regression analysis. The average duration of anaesthesia was 40 min longer for RATS than for VATS (*P* < .001). Marginal mean was 4.49 h under VATS and 5.19 h under RATS.

MD was +0.71 h (95% CI, +0.64 to +0.77) (approximately +42 min). No significant differences were observed in postoperative chest tube duration or hospital stay in multivariable analysis.

### Sensitivity analysis

1. The association between RATS and an increased risk of postoperative ventilation remained consistent when the endpoint definition was varied. The finding persisted when using a shorter time window and when restricting the outcome to re-initiation of invasive ventilation events only.
2. With additional adjustment for analgesic methods, the aIRR for ventilator use decreased but remained elevated among RATS (adjusted IRR 1.89, 95% CI, 1.31-2.73). After adjusting anaesthesia hours in addition to analgesic methods, the analysis remained similar results (aIRR 1.85, 95% CI, 1.28-2.67). As shown in **Figure 2**, the aIRR for ventilator use without adjusting for these 2 variables (total effect) was 1.96, 95% CI (1.36-2.81), indicating that the indirect effects of mediating analgesic methods and anaesthesia hours were small.
3. In the analysis stratified by year of surgery, although the overall risk of ventilation was significantly lower in 2020 compared to 2018, RATS was consistently associated with a higher risk of postoperative ventilation in each year.
4. Excluding early cases to account for the learning curve resulted in a reanalysis cohort of 1452 RATS cases. In this cohort, the aIRR for in-hospital mortality decreased (aIRR 0.41; 95% CI, 0.06-2.79), while the aIRR for postoperative mechanical ventilation remained elevated (aIRR 1.85; 95% CI, 0.91-3.78) (**Tables S2 and S3**).
5. In the primary analysis, the variances of the institution-level random effect were relatively high across many outcomes. For in-hospital death, τ^2^ was almost zero, because the incidence was very rare. For mechanical ventilation, τ^2^ was 0.39 (95% CI, 0.21-0.71). A caterpillar plot showing facility-level random intercepts for other outcomes is presented in **Figure S2**. This indicates that a significant proportion of the variation in postoperative ventilation management was attributable to differences between facilities rather than patient factors. To correct for this clustering effect, a sensitivity analysis restricted to 130 facilities (18 637 cases) performing both RATS and VATS was conducted. In this within-hospital subset, the facility variance decreased to 0.26 (95% CI, 0.10-0.65). Thus, when limited to facilities with mixed exposure composition, the between-hospital variation was small. In this analysis as well, the robot-assisted surgery group showed a significantly higher rate of mechanical ventilation (IRR 1.93; 95% CI, 1.32-2.83, *P* = .001), consistent with the main findings.
6. Analysis restricted to lobectomy and segmentectomy showed no material deviation from the primary analysis.
7. After multiple imputation of missing values, the distributions of covariates showed no material changes compared with the complete-case dataset. Instead of excluding 8434 cases with missing values for BMI, smoking history, disease stage, or surgical field, we included all 55 975 eligible cases. The 200 excluded cases were those not meeting the inclusion criteria (e.g., multiple surgeries). Multilevel Poisson regression after imputation produced results consistent with the main analysis (**Table S4**). Similar results were observed when changing the number of imputations in preliminary checks (*m* = 20, 60), consistent with the modest fraction of missing information.
8. As a result of limiting the analysis to specific surgical years and facilities, the sample size was 6309 cases (VATS = 4780, RATS = 1529, among 126 institutions). The 63 cases where the stabilized weight exceeded the 99th percentile were replaced with the 99th percentile value. The effective sample sizes were 4727 for VATS and 1374 for RATS, and remained high. Following IPW adjustment, standardized mean differences for most covariates improved to below 0.1, and variance ratios fell within acceptable ranges. Only resection type (lobectomy vs segmentectomy, 0.19) and stage (0.15) slightly exceeded the threshold. **Figures S3 and S4** show the plot of standardized mean differences for covariates (love plot) and the kernel density plot of weight distribution. Overall, covariate balance was favourable, and the results of weighted multilevel Poisson regression were similar to the main analysis. (**Table S5**).

## DISCUSSION

This study, using a large nationwide database, provides a real-world assessment of RATS’ perioperative safety during its early introduction in Japan. Our primary finding is that while overall perioperative safety, including in-hospital mortality, was comparable between RATS and VATS, RATS was associated with a statistically significant, though small in absolute terms, increase in the risk of postoperative mechanical ventilation.

Regarding our primary endpoint, in-hospital mortality, our multivariable analysis found no statistically significant difference between the groups. However, this finding should be interpreted with caution, as the very low event rate in both groups (approximately 0.3%) limited the statistical power to detect a true difference if one existed.

Notably, the direction of the point estimate in our primary analysis—suggesting a higher risk for RATS—contrasts with high-quality meta-analyses that report a significant protective effect for RATS (5). We hypothesize that this discrepancy stems from a combination of 2 factors unique to our real-world Japanese cohort. First, the baseline mortality rate for VATS in our study (0.30%) is exceptionally low compared to rates reported in the meta-analysis (1.22%), which can statistically magnify the relative impact of even a small number of events in the RATS group. Second, this effect is likely compounded by the inclusion of the early RATS adoption period. As shown in the sensitivity analysis, after accounting for the institutional learning curve, the point estimate for mortality shifted dramatically to favour RATS, aligning with the direction reported in previous literature. This suggests that the counterintuitive direction of our primary finding was driven by a small number of events during the learning curve, the effect of which was amplified by an extremely low baseline risk in the control group.

Among the secondary endpoints, the key finding was a statistically significant association between RATS and increased postoperative ventilation, though the absolute RD was small (VATS vs RATS; 0.67% vs 0.92%). This statistical significance is likely magnified by the exceptionally low baseline ventilation rate in our Japanese VATS cohort (0.7%), which contrasts sharply with rates near 5% in other cohorts.^10^ Furthermore, this association may be influenced by significant residual confounding.

The accuracy of the data for this finding is considered high, as procedures such as reintubation and mechanical ventilation duration are directly linked to reimbursement calculations within Japan’s DPC system.

The association between RATS and increased ventilation risk proved robust across a series of sensitivity analyses. The finding persisted after adjusting for potential mediators like analgesic methods, accounting for the institutional learning curve, varying the endpoint definition, and stratifying by year of surgery.

While the DPC data do not specify the causes of mechanical ventilation, existing literature offers several plausible mechanisms. First, the longer anaesthesia time observed for RATS in our study is a potential contributing factor. A previous study indicated that for every additional hour of anaesthesia, the risk of postoperative respiratory failure increases by a factor of 1.22 (95% CI, 1.04-1.44).^13^

Second, intraoperative ventilator management differences may play a role. A multicentre retrospective study reported that higher dynamic driving power—calculated as tidal volume (*V*_t_) × respiratory rate (RR) × 1/2 (plateau pressure [*P*_plat_] − positive end-expiratory pressure [PEEP])—was associated with an increased risk of postoperative reintubation.^14^ Furthermore, a prospective study found that RATS procedures involved significantly higher RR and *P*_plat_ compared to VATS.^15^ These findings may help explain the elevated risk of postoperative mechanical ventilation observed in our study.

To optimize outcomes, future multicentre studies should therefore focus on detailed procedural factors—including specific anaesthesia and ventilator management protocols—to elucidate the precise mechanisms behind this signal.

This study has several limitations inherent to the DPC database. First, outcomes are limited to the hospitalization period. Second, the database lacks granular clinical and surgical details, such as tumour size, specifics of lymph node dissection, conversion to thoracotomy, or individual surgeon experience. Third, significant unmeasured confounding from institutional-level variations in perioperative management likely exists. While we attempted to mitigate this with adjustments for anaesthesia method and a within-facility analysis, these cannot fully capture granular protocol differences. Fourth, we did not evaluate the impact of different robotic system generations. Fifth, although a sensitivity analysis using multiple imputation yielded results consistent with our primary findings (**Table S4**), our analysis was based on a complete-case approach, which excluded approximately 15% of potential cases due to missing data in key variables. This exclusion could introduce selection bias if the missingness is not completely at random, potentially limiting the generalizability of our findings. Lastly, its retrospective design limits causal inference, and prospective studies are needed to confirm these findings.

## CONCLUSION

This analysis of real-world data from the early introduction of RATS in Japan demonstrates that overall perioperative safety is comparable between RATS and VATS for lung cancer surgery. The key finding was a statistically significant association between the robotic approach and an increased risk of postoperative ventilation (marginal RD, +0.52 pp). This association remained consistent even after additional adjusting for concerning factors such as the learning curve, analgesic methods, and year of surgery. However, the absolute RD between the 2 groups was small, and it is plausible that different perioperative management strategies between facilities represent significant residual confounding. This suggests that future prospective studies should investigate specific procedural factors, including anaesthetic management, and patient selection to optimize outcomes.

## Supplementary Material

ivag005_Supplementary_Data

## Data Availability

The data underlying this article was provided by the DPC Research Group under license for the current study and cannot be shared publicly due to privacy and ethical restrictions. Data may be available from the corresponding author upon reasonable request and with permission of the DPC Research Group.
