# Supplementary material for: Robotic Versus Video-Assisted Thoracoscopic Lobectomy/Segmentectomy: Multilevel Analysis in Japan
Source: Interdiscip Cardiovasc Thorac Surg. 2026 Jan 9;41(1):ivag005. doi: 10.1093/icvts/ivag005 (PMC12854723; doi:10.1093/icvts/ivag005)
Supplement: ivag005_Supplementary_Data [file ivag005_supplementary_data.zip › Supplementary Table.docx]

| **Table S1. Comparison between postoperative outcomes and surgical procedures.** | | | | | | | | | | | | |
| --- | --- | --- | --- | --- | --- | --- | --- | --- | --- | --- | --- | --- |
|  | |  | | |  | VATS |  | RATS |  | Difference compared to VATS |  | P |
|  | |  | | |  | n=44,706 |  | n=2,835 |  |  |  |  |
| Outcomes | | | | |  | events, n (%) |  | events, n (%) |  | percentage points (pp) or mean difference (95% CI) |  |  |
| In‐hospital death | | | | | | 134 (0.3%) |  | 9 (0.3%) |  | +0.02pp (-0.20, +0.23) |  | 0.867 |
| Postoperative hospital stay (days), mean (SD) | | | | | | 9.12 (7.56) |  | 8.33 (9.04) |  | -0.8 days (-1.13, -0.42) |  | <0.001 |
| Anesthesia Time (hours), mean (SD) | | | | | | 4.46 (1.60) |  | 5.12 (1.55) |  | +0.7 hours (+0.59, +0.71) |  | <0.001 |
| Surgical-related endpoints | | | | | |  |  |  |  |  |  |  |
|  | | Red blood cell transfusion on the day of surgery | | | | 694 (1.6%) |  | 26 (0.9%) |  | -0.6pp (-1.00, -0.27) |  | 0.007 |
|  | | Red blood cell transfusion within 7 days | | | | 877 (2.0%) |  | 41 (1.5%) |  | -0.5pp (-0.97, -0.06) |  | 0.053 |
|  | | Intraoperative arrhythmia | | | | 359 (0.8%) |  | 10 (0.4%) |  | -0.4pp (-0.68, -0.22) |  | 0.008 |
|  | | Use of antibiotics after 5 days postoperatively | | | | 4,026 (9.0%) |  | 186 (6.6%) |  | -2.4pp (-3.39, -1.50) |  | <0.001 |
| Intraoperative, postoperative management | | | | | |  |  |  |  |  |  |  |
|  | | Ventilator fitted from day 1 to 30 | | | | 300 (0.7%) |  | 26 (0.9%) |  | +0.2pp (-0.11, +0.61) |  | 0.124 |
|  | | Postoperative thoracic drain insertion | | | | 42009 (94.0%) |  | 2628 (92.7%) |  | -1.3pp (-2.25, -0.29) |  | 0.006 |
|  | |  | | Duration of postoperative chest tube placement (days), mean (SD) | | 3.3 (2.7) |  | 3.2 (2.4) |  | -0.2 days (-0.27, -0.08) |  | <0.001 |
|  | | Thoracentesis or insertion of a thoracic drain within 30 days | | | | 2916 (6.5%) |  | 190 (6.7%) |  | +0.18pp (-0.77, +1.13) |  | 0.708 |
|  | | Performed Nerve block | | | | 5,338 (11.9%) |  | 541 (19.1%) |  | +7.1pp (+5.67, +8.62) |  | <0.001 |
|  | | Performed epidural anesthesia | | | | 28,970 (64.8%) |  | 1,438 (50.7%) |  | -14.1pp (-15.97, -12.19) |  | <0.001 |
|  | |  | | Duration of epidural anesthesia continuous administration (days), mean (SD) | | 1.6 (1.6) |  | 1.0 (1.3) |  | -0.3 days (-0.35, -0.24) |  | <0.001 |
| Reoperation for any of the complications | | | | | |  |  |  |  |  |  |  |
|  | | Bleeding | | |  | 50 (0.1%) |  | 4 (0.1%) |  | +0.03pp (-0.11, +0.17) |  | 0.654 |
|  | | Pyothorax | | | | 57 (0.1%) |  | 0 (0.0%) |  | -0.13pp (-0.16, -0.09) |  | 0.057 |
|  | | Pulmonary fistula | | | | 164 (0.4%) |  | 9 (0.3%) |  | -0.05pp (-0.26, +0.17) |  | 0.672 |
|  | | Bronchial fistula | | | | 38 (0.1%) |  | 1 (0.0%) |  | -0.05pp (-0.12, +0.02) |  | 0.370 |
|  | | Chylothorax | | | | 26 (0.1%) |  | 2 (0.1%) |  | +0.01pp (-0.09, +0.11) |  | 0.792 |
| Postoperative complication | | | | | |  |  |  |  |  |  |  |
|  | | Pulmonary fistula | | | | 2,470 (5.5%) |  | 92 (3.2%) |  | -2.3pp (-2.97, -1.59) |  | <0.001 |
|  | | Bronchial fistula | | | | 24 (0.1%) |  | 1 (0.0%) |  | -0.02pp (-0.09, +0.05) |  | 0.678 |
|  | | Chylothorax | | | | 154 (0.3%) |  | 10 (0.4%) |  | +0.008pp (-0.22, +0.23) |  | 0.942 |
|  | | Acute lung injury | | | | 387 (0.5%) |  | 7 (0.2%) |  | -0.6pp (-0.82, -0.42) |  | <0.001 |
|  | | Pulmonary embolism | | | | 89 (0.2%) |  | 6 (0.2%) |  | +0.01pp (-0.16, 0.19) |  | 0.885 |
|  | | Interstitial pneumonia | | | | 144 (0.3%) |  | 11 (0.4%) |  | +0.07pp (-0.17, +0.30) |  | 0.551 |
|  | | Pneumonia | | | | 3,959 (8.9%) |  | 181 (6.4%) |  | -2.5pp (-3.40, -1.53) |  | <0.001 |
|  | | Respiratory insufficiency | | | | 1,129 (2.5%) |  | 96 (3.4%) |  | +0.9pp (+0.18, +1.54) |  | 0.005 |
|  | | Arrhythmia | | | | 1,089 (2.4%) |  | 65 (2.3%) |  | -0.1pp (-0.71, +0.43) |  | 0.631 |
|  | | Postoperative pain | | | | 16,061 (35.9%) |  | 937 (33.1%) |  | -2.9pp (-4.67, -1.09) |  | 0.002 |
|  | | Wound dehiscence or infection | | | | 262 (0.6%) |  | 15 (0.5%) |  | -0.06pp (-0.33, +0.22) |  | 0.699 |
|  |  | | CI, Confidence Interval; SD, Standard Deviation; RATS, Robotic-Assisted Thoracoscopic Surgery; VATS, Video-Assisted Thoracoscopic Surgery | | | | | | | | | |

| Table S2. Association between the postoperative outcomes and kind of surgical procedure as estimated based on a multilevel poisson regression analysis with robust variance; excluding 20 RATS cases from each center in descending order of operative date | | | | | | | | | | | | | | | |
| --- | --- | --- | --- | --- | --- | --- | --- | --- | --- | --- | --- | --- | --- | --- | --- |
|  |  |  |  |  | Age-sex adjusted | | | |  | Multivariate* | | | | |  |
| Outcomes | Operation procedure | Number of each surgery | Number of events | % of events | Incidence rate ratio | 95%  Confidence Interval | | P |  | Incidence rate ratio | 95%  Confidence Interval | | P | |  |
| In‐hospital death | VATS | 44,706 | 134 | 0.3% | reference |  |  |  |  | reference |  |  | |  |  |
|  | RATS | 1,452 | 1 | 0.1% | 0.28 | 0.04 | 1.90 | 0.193 |  | 0.41 | 0.06 | 2.79 | | 0.363 |  |
|  |  |  |  |  |  |  |  |  |  |  |  |  | |  |  |
| Ventilator postoperatively from day 1 to 30 | VATS | 44,706 | 300 | 0.7% | reference |  |  |  |  | reference |  |  | |  |  |
|  | RATS | 1,452 | 11 | 0.8% | 1.30 | 0.65 | 2.60 | 0.450 |  | 1.85 | 0.91 | 3.78 | | 0.091 |  |
|  |  |  |  |  |  |  |  |  |  |  |  |  | |  |  |
| Reoperation for any of the complications | VATS | 44,706 | 324 | 0.7% | reference |  |  |  |  | reference |  |  | |  |  |
|  | RATS | 1,452 | 8 | 0.6% | 0.97 | 0.45 | 2.10 | 0.944 |  | 1.07 | 0.48 | 2.35 | | 0.876 |  |
|  |  |  |  |  |  |  |  |  |  |  |  |  | |  |  |
| Pulmonary complications† | VATS | 44,706 | 5534 | 12.4% | reference |  |  |  |  | reference |  |  | |  |  |
|  | RATS | 1,452 | 144 | 9.9% | 1.07 | 0.81 | 1.42 | 0.638 |  | 1.07 | 0.81 | 1.41 | | 0.642 |  |
|  |  |  |  |  |  |  |  |  |  |  |  |  | |  |  |
| Pulmonary fistula or bronchial fistula | VATS | 44,706 | 2487 | 5.6% | reference |  |  |  |  | reference |  |  | |  |  |
|  | RATS | 1,452 | 43 | 3.0% | 0.77 | 0.60 | 0.99 | 0.042 |  | 0.79 | 0.62 | 1.01 | | 0.062 |  |
|  |  |  |  |  |  |  |  |  |  |  |  |  | |  |  |
| Postoperative pain | VATS | 44,706 | 16061 | 35.9% | reference |  |  |  |  | reference |  |  | |  |  |
|  | RATS | 1,452 | 494 | 34.0% | 1.08 | 0.96 | 1.21 | 0.209 |  | 1.08 | 0.97 | 1.21 | | 0.164 |  |
|  |  |  |  |  |  |  |  |  |  |  |  |  | |  |  |
| Postoperative thoracentesis or continuous thoracic drainage | VATS | 44,706 | 2916 | 6.5% | reference |  |  |  |  | reference |  |  | |  |  |
|  | RATS | 1,452 | 86 | 5.9% | 1.11 | 0.84 | 1.47 | 0.462 |  | 1.10 | 0.83 | 1.44 | | 0.508 |  |
|  |  |  |  |  |  |  |  |  |  |  |  |  | |  |  |
| Red blood cell transfusion on the day of surgery | VATS | 44,706 | 694 | 1.6% | reference |  |  |  |  | reference |  |  | |  |  |
|  | RATS | 1,452 | 10 | 0.7% | 0.60 | 0.35 | 1.02 | 0.059 |  | 0.69 | 0.40 | 1.19 | | 0.184 |  |
|  |  |  |  |  |  |  |  |  |  |  |  |  | |  |  |
| Red blood cell transfusion from the day of surgery to 7 days postoperatively | VATS | 44,706 | 877 | 2.0% | reference |  |  |  |  | reference |  |  | |  |  |
|  | RATS | 1,452 | 17 | 1.2% | 0.78 | 0.51 | 1.18 | 0.235 |  | 0.87 | 0.57 | 1.34 | | 0.534 |  |
|  |  |  |  |  |  |  |  |  |  |  |  |  | |  |  |
| Antibiotics from 5 days postoperatively to 14 days postoperatively | VATS | 44,706 | 4026 | 9.0% | reference |  |  |  |  | reference |  |  | |  |  |
|  | RATS | 1,452 | 97 | 6.7% | 0.85 | 0.61 | 1.20 | 0.352 |  | 0.90 | 0.65 | 1.24 | | 0.507 |  |
| * Adjusted for sex, age, Body Mass Index, smoking, cancer stage, coexisting diseases, lobectomy or segmentectomy, area of surgery, number of surgeries and fiscal year of day of operation  † Pulmonary complications were defined as a composite endpoint including acute lung injury, pulmonary embolism, interstitial pneumonia, pneumonia, and respiratory insufficiency, identified by their respective ICD-10 codes (see Appendix A) | | | | | | | | | | | | | | | |
| Abbreviations: VATS, video-assisted thoracoscopic surgery; RATS, robotic-assisted thoracoscopic surgery. | | | | | | | | | | | | | | | |

| Table S3. Mean difference in the operation time, postoperative recovery regarding surgical procedure as estimated based on a multilevel linear regression analysis; excluding 20 RATS cases from each center in descending order of operative date | | | | | | | | | | | | | |
| --- | --- | --- | --- | --- | --- | --- | --- | --- | --- | --- | --- | --- | --- |
|  |  |  |  | Age-sex adjusted | | | |  | Multivariate* | | | | |
| Outcomes | Operation procedure | Mean | Standard Error | Coefficient | 95%  Confidence Interval | | P |  | Coefficient | 95%  Confidence Interval | | P | |
| Length of anesthesia (hours) | VATS | 4.46 | 0.01 | reference |  |  |  |  | reference |  |  | |  |
|  | RATS | 4.86 | 0.04 | 0.48 | 0.39 | 0.56 | < 0.001 |  | 0.50 | 0.41 | 0.59 | | < 0.001 |
|  |  |  |  |  |  |  |  |  |  |  |  | |  |
| Length of days of postoperative chest drain placement† | VATS | 3.32 | 0.01 | reference |  |  |  |  | reference |  |  | |  |
|  | RATS | 3.20 | 0.06 | 0.03 | -0.13 | 0.19 | 0.758 |  | 0.07 | -0.09 | 0.23 | | 0.391 |
|  |  |  |  |  |  |  |  |  |  |  |  | |  |
| Length of postoperative hospital stay‡ (days) | VATS | 9.12 | 0.04 | reference |  |  |  |  | reference |  |  | |  |
|  | RATS | 8.40 | 0.30 | -0.17 | -0.52 | 0.39 | 0.775 |  | 0.30 | -0.15 | 0.76 | | 0.192 |
| *Adjusted for sex, age, Body Mass Index, smoking, cancer stage, coexisting diseases, lobectomy or segmentectomy, area of surgery, number of surgeries and fiscal year of day of operation | | | | | | | | | | | | | |
| † Excluding no record of drain | | | | | | | | | | | | | |
| ‡ Excluding deaths during hospitalization | | | | | | | | | | | | | |
| Abbreviations: VATS, video-assisted thoracoscopic surgery; RATS, robotic-assisted thoracoscopic surgery. | | | | | | | | | | | | | |

| Table S4. Association between the postoperative outcomes and kind of surgical procedure as estimated based on a multilevel poisson regression analysis with robust variance after multiple imputation | | | | | | | | | | | | | | | |
| --- | --- | --- | --- | --- | --- | --- | --- | --- | --- | --- | --- | --- | --- | --- | --- |
|  |  |  |  |  | Age-sex adjusted | | | |  | Multivariate* | | | | |  |
| Outcomes | Operation procedure | Number of each surgery | Number of events | % of events | Incidence rate ratio | 95%  Confidence Interval | | P |  | Incidence rate ratio | 95%  Confidence Interval | | P | |  |
| In‐hospital death | VATS | 52,626 | 196 | 0.4% | reference |  |  |  |  | reference |  |  | |  |  |
|  | RATS | 3.349 | 12 | 0.4% | 1.46 | 0.82 | 2.62 | 0.198 |  | 1.48 | 0.82 | 2.67 | | 0.190 |  |
|  |  |  |  |  |  |  |  |  |  |  |  |  | |  |  |
| Ventilator postoperatively from day 1 to 30 | VATS | 52,626 | 412 | 0.8% | reference |  |  |  |  | reference |  |  | |  |  |
|  | RATS | 3,349 | 31 | 0.9% | 1.68 | 1.20 | 2.35 | 0.003 |  | 1.60 | 1.14 | 2.25 | | 0.006 |  |
|  |  |  |  |  |  |  |  |  |  |  |  |  | |  |  |
| Reoperation for any of the complications | VATS | 52,626 | 401 | 0.8% | reference |  |  |  |  | reference |  |  | |  |  |
|  | RATS | 3,349 | 19 | 0.6% | 0.90 | 0.56 | 1.46 | 0.675 |  | 0.87 | 0.53 | 1.44 | | 0.593 |  |
|  |  |  |  |  |  |  |  |  |  |  |  |  | |  |  |
| Pulmonary complications† | VATS | 52,626 | 6384 | 12.1% | reference |  |  |  |  | reference |  |  | |  |  |
|  | RATS | 3,349 | 344 | 10.3% | 0.86 | 0.58 | 1.25 | 0.425 |  | 0.98 | 0.84 | 1.13 | | 0.752 |  |
|  |  |  |  |  |  |  |  |  |  |  |  |  | |  |  |
| Pulmonary fistula or bronchial fistula | VATS | 52,626 | 2832 | 5.4% | reference |  |  |  |  | reference |  |  | |  |  |
|  | RATS | 3,349 | 108 | 3.2% | 0.65 | 0.28 | 1.48 | 0.304 |  | 0.79 | 0.60 | 1.04 | | 0.093 |  |
|  |  |  |  |  |  |  |  |  |  |  |  |  | |  |  |
| Postoperative pain | VATS | 52,626 | 18667 | 35.5% | reference |  |  |  |  | reference |  |  | |  |  |
|  | RATS | 3,349 | 1057 | 31.6% | 0.87 | 0.67 | 1.14 | 0.320 |  | 1.07 | 1.00 | 1.14 | | 0.044 |  |
|  |  |  |  |  |  |  |  |  |  |  |  |  | |  |  |
| Postoperative thoracentesis or continuous thoracic drainage | VATS | 52,626 | 3499 | 6.6% | reference |  |  |  |  | reference |  |  | |  |  |
|  | RATS | 3,349 | 245 | 7.3% | 1.24 | 0.94 | 1.63 | 0.127 |  | 1.07 | 0.93 | 1.23 | | 0.361 |  |
|  |  |  |  |  |  |  |  |  |  |  |  |  | |  |  |
| Red blood cell transfusion on the day of surgery | VATS | 52,626 | 839 | 1.6% | reference |  |  |  |  | reference |  |  | |  |  |
|  | RATS | 3,349 | 35 | 1.0% | 0.95 | 0.64 | 1.40 | 0.795 |  | 1.12 | 0.79 | 1.60 | | 0.521 |  |
|  |  |  |  |  |  |  |  |  |  |  |  |  | |  |  |
| Red blood cell transfusion from the day of surgery to 7 days postoperatively | VATS | 52,626 | 1068 | 2.0% | reference |  |  |  |  | reference |  |  | |  |  |
|  | RATS | 3,349 | 53 | 1.6% | 1.08 | 0.77 | 1.51 | 0.672 |  | 1.17 | 0.88 | 1.55 | | 0.276 |  |
|  |  |  |  |  |  |  |  |  |  |  |  |  | |  |  |
| Antibiotics from 5 days postoperatively to 14 days postoperatively | VATS | 52,626 | 3464 | 6.6% | reference |  |  |  |  | reference |  |  | |  |  |
|  | RATS | 3,349 | 155 | 4.6% | 0.89 | 0.63 | 1.25 | 0.504 |  | 0.85 | 0.70 | 1.03 | | 0.092 |  |
| * Adjusted for sex, age, Body Mass Index, smoking, cancer stage, coexisting diseases, lobectomy or segmentectomy, area of surgery, number of surgeries and fiscal year of day of operation  † Pulmonary complications were defined as a composite endpoint including acute lung injury, pulmonary embolism, interstitial pneumonia, pneumonia, and respiratory insufficiency, identified by their respective ICD-10 codes (see Appendix A) | | | | | | | | | | | | | | | |
| Abbreviations: VATS, video-assisted thoracoscopic surgery; RATS, robotic-assisted thoracoscopic surgery. | | | | | | | | | | | | | | | |

| Table S5. Association between the postoperative outcomes and kind of surgical procedure as estimated based on a multilevel poisson regression analysis with inversed probability weighting | | | | | | | | | | | | | | |
| --- | --- | --- | --- | --- | --- | --- | --- | --- | --- | --- | --- | --- | --- | --- |
|  | Operation procedure | Number of each surgery | Number of events | % of events | Age-sex adjusted | | | |  | Multivariable† | | | | |
| Outcomes |  |  |  |  | Incidence rate ratio | 95%  Confidence Interval | | *p* |  | Incidence rate ratio | 95%  Confidence Interval | | *p* | |
| Deaths |  |  |  |  |  |  |  |  |  |  |  |  |  | |
|  | VATS | 4,780 | 7 | 0.2% | reference |  |  |  |  | reference |  |  |  | |
|  | RATS | 1,529 | 4 | 0.3% | 1.80 | 0.58 | 5.59 | 0.312 |  | 2.35 | 0.71 | 7.71 | 0.160 | |
| Ventilator within 30 days after surgery |  |  |  |  |  |  |  |  |  |  |  |  |  | |
|  | VATS | 4,780 | 19 | 0.4% | reference |  |  |  |  | reference |  |  |  | |
|  | RATS | 1,529 | 11 | 0.7% | 1.86 | 0.87 | 3.95 | 0.107 |  | 2.20 | 1.05 | 4.60 | 0.036 | |
| Reoperation for any of the complications |  |  |  |  |  |  |  |  |  |  |  |  |  | |
|  | VATS | 4,780 | 41 | 0.9% | reference |  |  |  |  | reference |  |  |  | |
|  | RATS | 1,529 | 3 | 0.2% | 0.23 | 0.05 | 1.03 | 0.054 |  | 0.30 | 0.07 | 1.29 | 0.107 | |
| Pulmonary complications |  |  |  |  |  |  |  |  |  |  |  |  |  | |
|  | VATS | 4,780 | 655 | 13.7% | reference |  |  |  |  | reference |  |  |  | |
|  | RATS | 1,529 | 141 | 9.2% | 0.96 | 0.81 | 1.14 | 0.642 |  | 1.00 | 0.84 | 1.19 | 0.983 | |
| Pulmonary fistula or bronchial fistula |  |  |  |  |  |  |  |  |  |  |  |  |  | |
|  | VATS | 4,780 | 365 | 7.6% | reference |  |  |  |  | reference |  |  |  | |
|  | RATS | 1,529 | 54 | 3.5% | 0.69 | 0.50 | 0.96 | 0.027 |  | 0.70 | 0.49 | 0.99 | 0.042 | |
| Postoperative pain |  |  |  |  |  |  |  |  |  |  |  |  |  | |
|  | VATS | 4,780 | 1458 | 30.5% | reference |  |  |  |  | reference |  |  |  | |
|  | RATS | 1,529 | 460 | 30.1% | 1.03 | 0.95 | 1.10 | 0.500 |  | 1.02 | 0.95 | 1.10 | 0.526 | |
| Postoperative thoracentesis or continuous thoracic drainage |  |  |  |  |  |  |  |  |  |  |  |  |  | |
|  | VATS | 4,780 | 317 | 6.6% | reference |  |  |  |  | reference |  |  |  | |
|  | RATS | 1,529 | 103 | 6.7% | 0.98 | 0.77 | 1.24 | 0.866 |  | 1.00 | 0.78 | 1.27 | 0.975 | |
| Red blood cell transfusion on the day of surgery |  |  |  |  |  |  |  |  |  |  |  |  |  | |
|  | VATS | 4,780 | 73 | 1.5% | reference |  |  |  |  | reference |  |  |  | |
|  | RATS | 1,529 | 13 | 0.9% | 0.71 | 0.40 | 1.25 | 0.236 |  | 0.87 | 0.47 | 1.62 | 0.669 | |
| Red blood cell transfusion from the day of surgery to 7 days postoperatively |  |  |  |  |  |  |  |  |  |  |  |  |  | |
|  | VATS | 4,780 | 92 | 1.9% | reference |  |  |  |  | reference |  |  |  | |
|  | RATS | 1,529 | 19 | 1.2% | 0.78 | 0.46 | 1.33 | 0.37 |  | 0.93 | 0.53 | 1.61 | 0.790 | |
| Antibiotics from 5 days postoperatively to 14 days postoperatively |  |  |  |  |  |  |  |  |  |  |  |  |  | |
|  | VATS | 4,780 | 370 | 7.7% | reference |  |  |  |  | reference |  |  |  | |
|  | RATS | 1,529 | 83 | 5.4% | 0.68 | 0.51 | 0.90 | 0.007 |  | 0.82 | 0.61 | 1.09 | 0.173 | |
| *Analysis restricted to cases operated on during the final year of the observation period and cases operated on at facilities that performed both VATS and RATS during the observation period. | | | | | | | | | | | | | |  |
| †A weighted Poisson regression model was fitted using stabilized inverse probability weights (IPW), where propensity scores for assignment to the RATS group were estimated with the same covariates as in the main multivariable analysis. Weights were stabilized and capped at the top 1%. | | | | | | | | | | | | | | |
| Abbreviations: VATS, video-assisted thoracoscopic surgery; RATS, robotic-assisted thoracoscopic surgery. | | | | | | | | | | | | | | |
