# Supplementary material for: Robotic Versus Video-Assisted Thoracoscopic Lobectomy/Segmentectomy: Multilevel Analysis in Japan
Source: Interdiscip Cardiovasc Thorac Surg. 2026 Jan 9;41(1):ivag005. doi: 10.1093/icvts/ivag005 (PMC12854723; doi:10.1093/icvts/ivag005)
Supplement: ivag005_Supplementary_Data [file ivag005_supplementary_data.zip › Supplementary Appendix.docx]

Appendix. Code Definitions for Comorbidities, Complications, and Interventions

Preoperative Comorbidities

| Comorbidity | ICD-10 Code |
| --- | --- |
| Chronic obstructive pulmonary disease | J43.0, J43.1, J43.2, J43.9, J44.0, J44.8, J44.9 |
| Interstitial pneumonia | J70.2, J70.3, J70.4, J84.1, J84.9, M05.1-J99.0, M32.1-J99.1, M33.0-J99.1, M33.1-J99.1, M33.2-J99.1, M35.1-J99.1 |
| Pulmonary hypertension | I27.0, I27.1, I27.2 |
| Respiratory insufficiency | J96.09, J96.10, J96.11, J96.19 |
| Chronic kidney disease | N18.1, N18.2, N18.3, N18.4, N18.5, N18.9 |
| Hypertension | I10, I11, I12, I15, I11.0, I12.0, I12.9, I13.9, I15.0, I15.1 |
| Diabetes | E10, E11, E12, E13, E14 |
| Coronary artery disease | I21.0, I21.1, I21.2, I21.3, I21.4, I21.9, I22.0, I22.1, I22.8, I22.9, I25.2 |
| Heart failure | I09.9, I11.0, I50.0, I50.1, I50.9 |
| Arrhythmia | I47.0, I48.0, I48.1, I48.2, I48.9, I49.8, R00.0 |
| Cerebral infarction | G45, I60, I61, I62, I63, I64, I65, I66, I67, I68 |
| Peripheral vascular disease | I80.0, I80.1, I80.2, I80.3, I80.8, I80.9, I82.0, I82.1, I82.2, I82.3, I82.8, I82.9, I83.9, I86.8 |

Postoperative Complications

| Complication | ICD-10 Code |
| --- | --- |
| Pulmonary fistula | J98.4 |
| Bronchial fistula | J98.0 |
| Chylothorax | I89.8, T81.2 |
| Acute lung injury | J80, J81, J70.8, J95.3, J95.9, J98.4 |
| Pulmonary embolism | I26.9 |
| Interstitial pneumonia | J70.2, J70.3, J70.4, J84.1, J84.9, M05.1-J99.0, M32.1-J99.1, M33.0-J99.1, M33.1-J99.1, M33.2-J99.1, M35.1-J99.1 |
| Pneumonia | J15, J20, J21, J22, J40, J18.0, J18.1, J18.8, J18.9, J20.9, J69.0, J95.4, J95.8 |
| Respiratory insufficiency | J95.8, J96.09, J96.10, J96.11, J96.19 |
| Arrhythmia | I47.0, I48.0, I48.1, I48.2, I48.9, I49.8, R00.0 |
| Postoperative pain | G58.0, R52.0, R52.1, R52.9, T81.8 |
| Wound dehiscence or infection | T81.0, T81.3, T81.4 |

Postoperative Interventions and Reoperations

| General Category | The Japanese medical intervention classification master code |
| --- | --- |
| Mechanical Ventilation | J045-00 |
| Postoperative thoracic drain insertion | J002-00 |
| Thoracentesis or insertion of a thoracic drain | J019-00, J008-00, D4192 |
| Nerve block | L008, L009 |
| Epidural anesthesia | L008-00, L003-00 |
| Reoperation for Bleeding | K494 |
| Reoperation for Pyothorax | K4962, K496_2, K496_4, K496_31, K496_32, K496_5, K507 |
| Reoperation for Pulmonary fistula | K513_04, K517_00 |
| Reoperation for Bronchial fistula | K509_04, K516_00 |
| Reoperation for Chylothorax | K501_00, K501_03 |
