# Supplementary material for: Robotic Versus Video-Assisted Thoracoscopic Lobectomy/Segmentectomy: Multilevel Analysis in Japan
Source: Interdiscip Cardiovasc Thorac Surg. 2026 Jan 9;41(1):ivag005. doi: 10.1093/icvts/ivag005 (PMC12854723; doi:10.1093/icvts/ivag005)
Supplement: ivag005_Supplementary_Data [file ivag005_supplementary_data.zip › Supplementary Figure.pptx]

## Slide 1
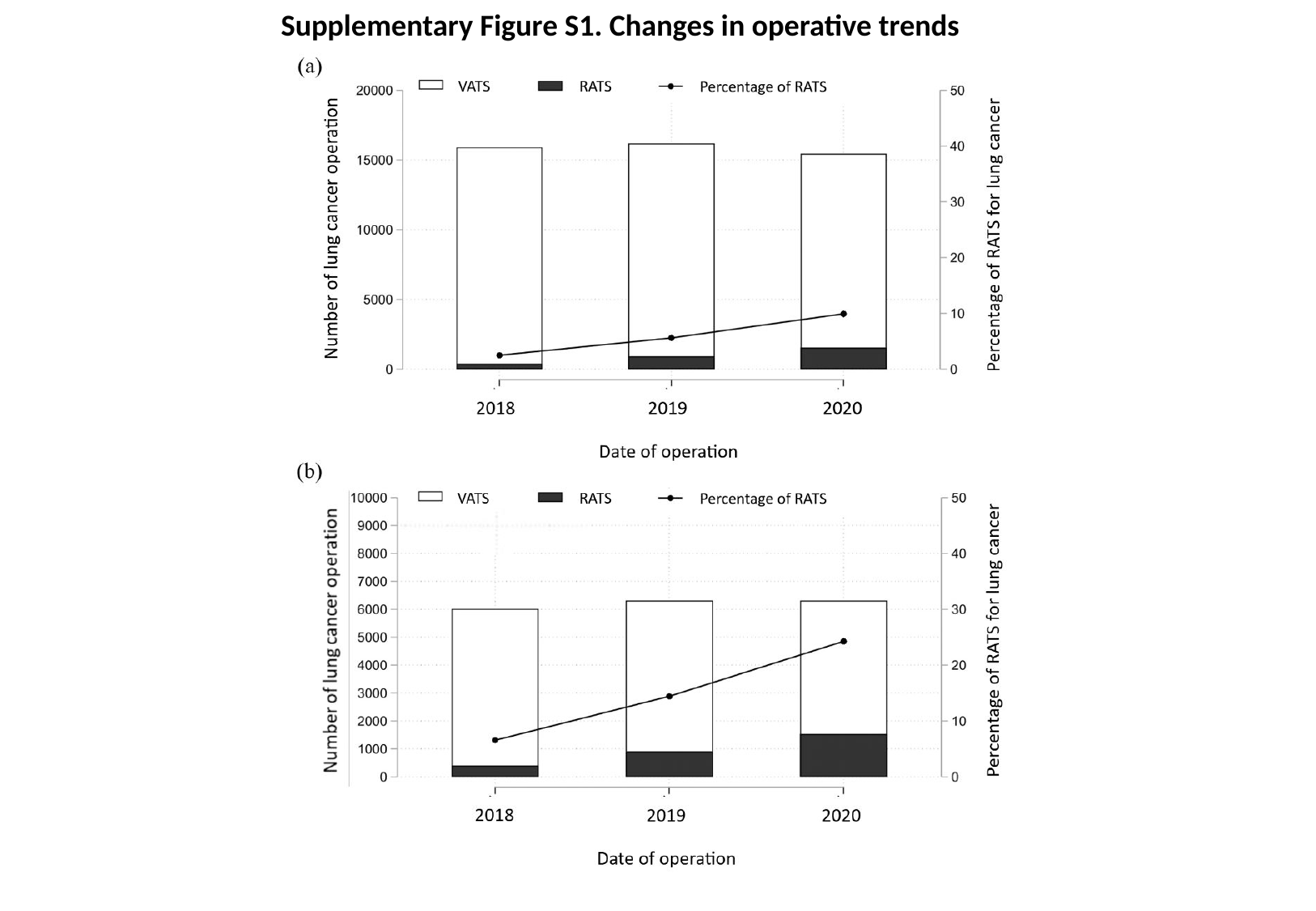

Supplementary Figure S1. Changes in operative trends

## Slide 2
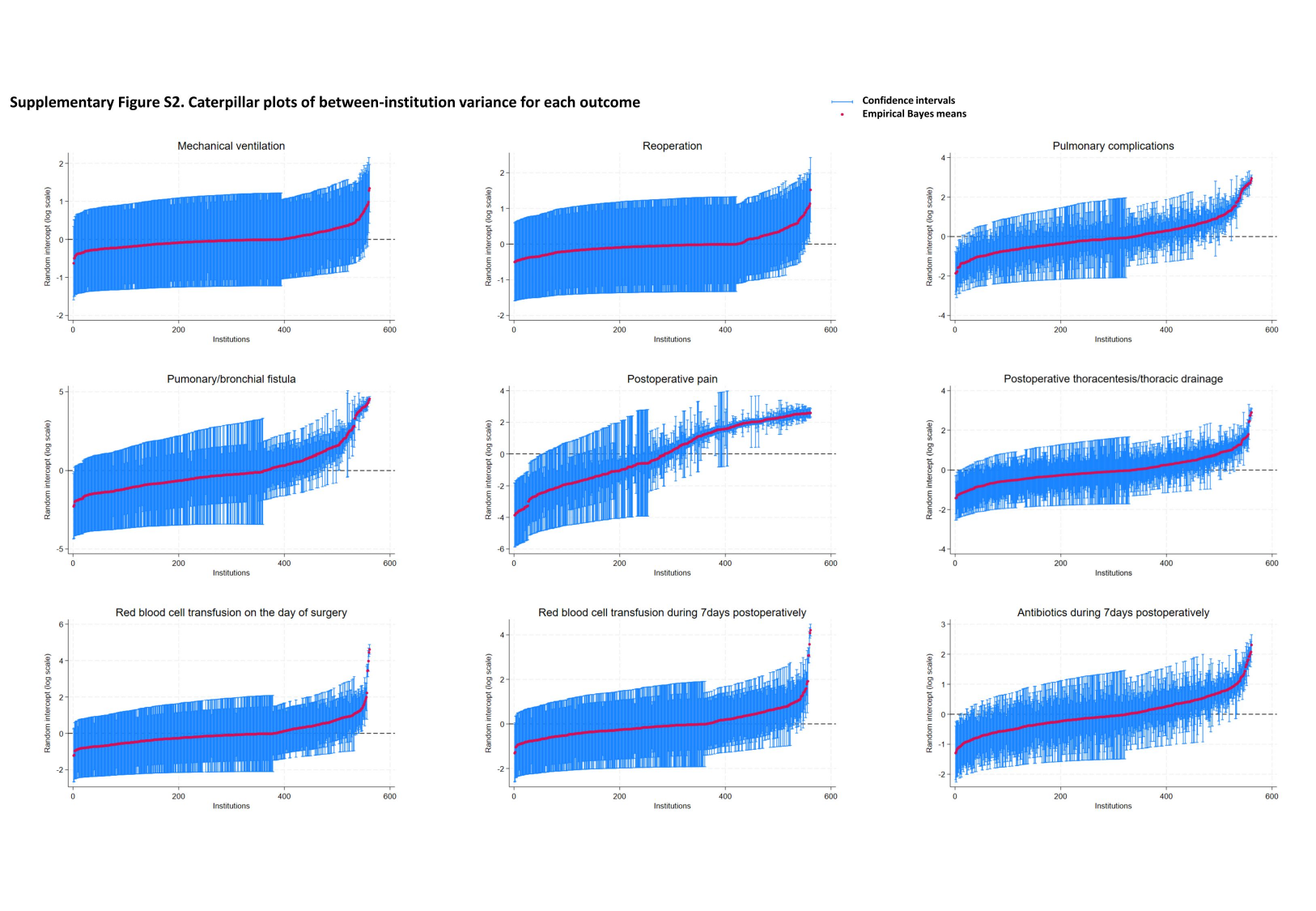

## Slide 3
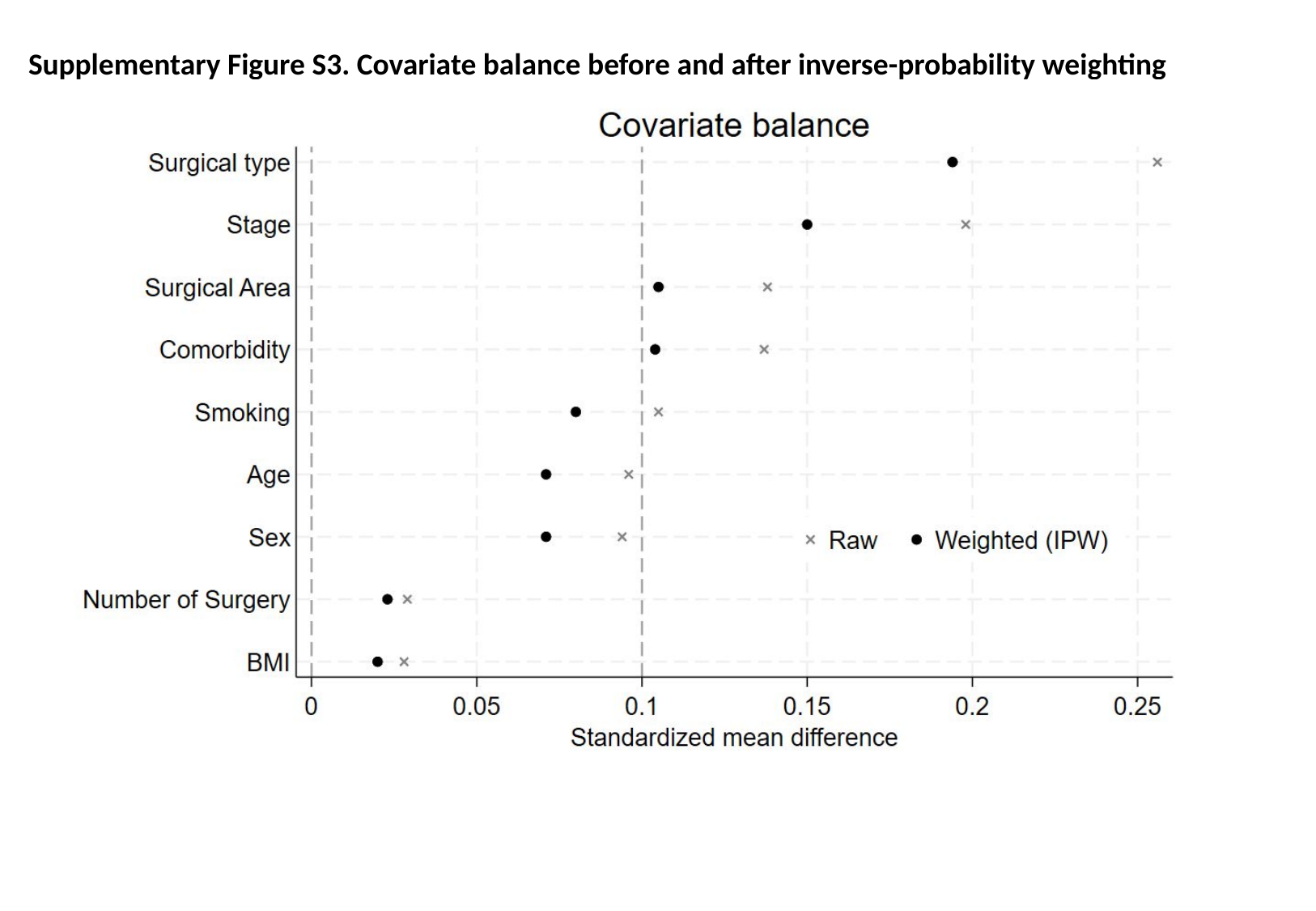

Supplementary Figure S3. Covariate balance before and after inverse-probability weighting

## Slide 4
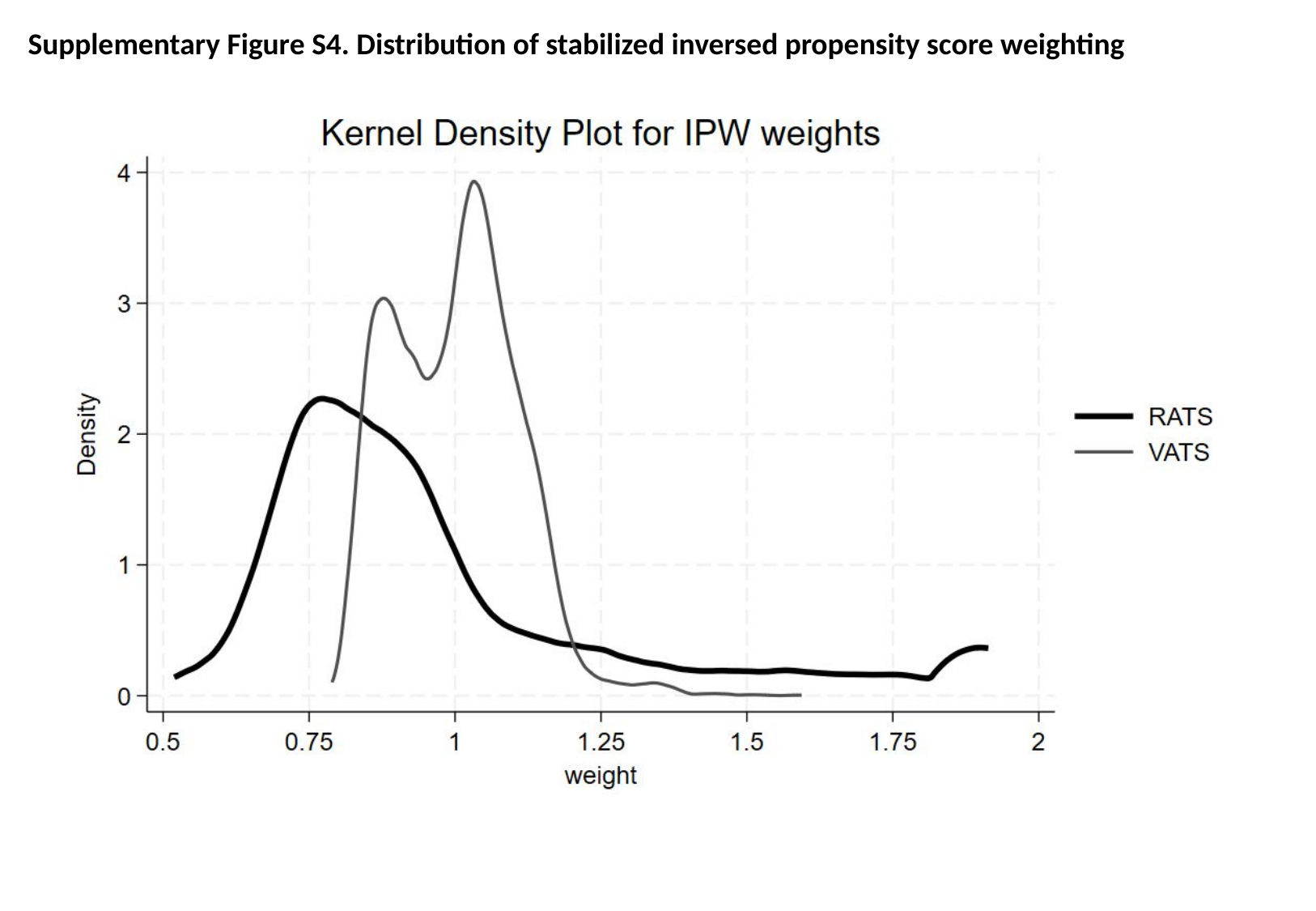

Supplementary Figure S4. Distribution of stabilized inversed propensity score weighting
